# Foreign body granulomatous reactions to cosmetic fillers

**DOI:** 10.4317/jced.50868

**Published:** 2012-10-01

**Authors:** Laura Carlos-Fabuel, Cristina Marzal-Gamarra, Silvia Martí-Álamo, Aisha Mancheño-Franch

**Affiliations:** 1 DDS. Master in Oral Medicine and Surgery. University of Valencia. Valencia (Spain).

## Abstract

Introduction: The use of different facial cosmetic fillers has increased in recent years. The introduction of apparently inert substances in the epidermis can give rise to foreign body granulomatous reactions. Objetives: A literature review is made of the foreign body granulomatous reactions to cosmetic fillers. Material and Methods: A PubMed-Medline search was made using the following keywords: “granulomatous reactions”, “foreign body reactions”, “esthetic fillers”, “cosmetic fillers”. The search was limited to articles published in English and Spanish during the last 10 years. A total of 22 articles were reviewed. Results: A great variety of substances have been found to give rise to foreign body granulomatous reactions. The most common locations are the upper and lower lip and the nasogenian sulcus. The clinical presentation is variable and can range from single or multiple nodules to diffuse facial swelling of hard-elastic consistency, accompanied by reddening. Most lesions are asymptomatic or cause only mild discomfort. The literature describes different treatments, including systemic corticosteroids, local tacrolimus infiltrations, minocycline, retinoids, allopurinol, 5% imiquimod, and surgical removal. Conclusions: In view of the current demand for esthetic treatments, the use of cosmetic fillers can be expected to increase in future, together with the incidence of complications.

** Key words:**Esthetic fillers, granulomatous reactions, foreign body reactions, cosmetic fillers.

## Introduction

The use of different facial cosmetic fillers has increased in recent years (1-8). However, the introduction of apparently inert substances in the epidermis can give rise to foreign body granulomatous reactions, among other undesirable effects (1,2,5). This has led to the development of many new filler materials. However, despite the incorporated improvements, which have contributed to reduce the number of complications, no ideal product has been developed to date. Such an ideal product should be biocompatible (i.e., with a low foreign body reaction risk), easy to inject, non-allergenic, inert and unable to migrate or displace after injection (1,9-12).

Different filler classifications have been proposed, according to the origin, durability and biodegradability of the product. In this context, filler materials can be classified as reabsorbable (i.e., those in which tissue volume ex-pansion after injection lasts a maximum of 4-6 months), semi-permanent (lasting about 18 months) or permanent (i.e., materials that cannot be eliminated)(3,4,7-9,11-13).  Table 1 describes the materials that are currently available and their classification according to the durability of the filling effect.

**Table 1:**
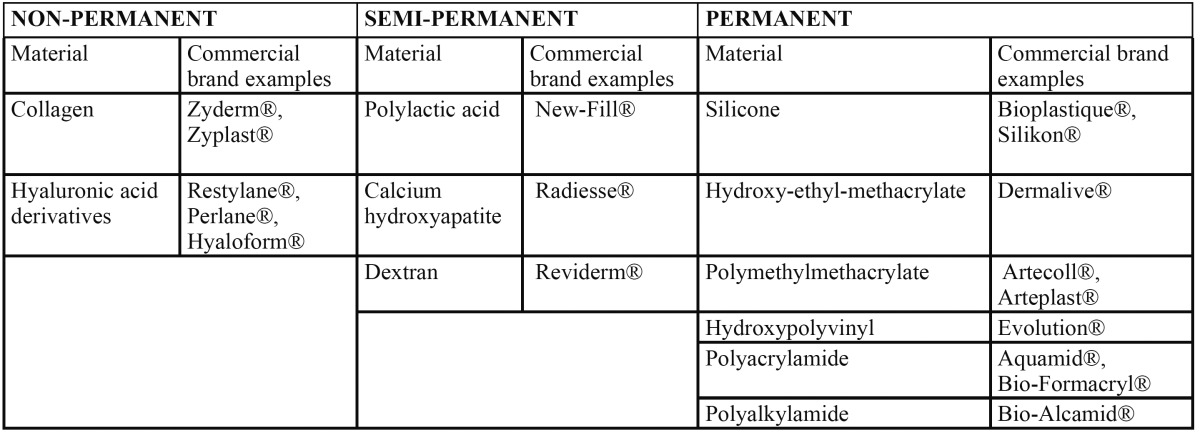
Filler materials are currently available and their classification according to the durability of the filling effect.

## Objectives

The present study offers a literature review of the foreign body granulomatous reactions to cosmetic fillers.

## Material and Methods

A PubMed-Medline literature search was made using the following keywords: *“granulomatous reactions”, “foreign body reactions”, “esthetic fillers”, “cosmetic fillers”*.

The search was limited to articles published in English and Spanish during the last 10 years.

A total of 22 articles were reviewed: one cross-sectional study, a cohort study, 9 clinical case series, 9 clinical cases and two review articles.

## Results

A great variety of substances have been found to give rise to foreign body granulomatous reactions. The most popular include: silicone, collagen, hyaluronic acid either alone or with acrylic gel (Dermalive®), polymethyl-methacrylate (Artecoll®, Arteplast®), polylactic acid, hydroxyapatite and polyalkylamide (Bio-Alcamid®). The most common locations are the upper and lower lip and the nasogenian sulcus. The clinical presentation is variable and can range from single or multiple nodules to diffuse facial swelling of hard-elastic consistency, accompanied by reddening. Most lesions are asymptomatic or cause only mild discomfort. The literature describes different treatments, including systemic corticosteroids (prednisone), local tacrolimus infiltrations, minocycline, retinoids (isotretinoin), allopurinol, 5% imiquimod, and surgical removal.

## Discussion

Independently of the filler material used, the mechanism of action and purpose are similar in all cases. The injected product induces a reaction of the surrounding connective tissue, with the depositing of more or less stable collagen that persists independently of phagocytosis of the filler material. The underlying mechanism of action remains unclear, though infection has been suggested to trigger an immune cross-reaction, or alternatively delayed immunity may be induced. In all cases, the objective of the filler material is to expand soft tissue volume.

Foreign body granulomatous reactions develop after a variable period of time ranging from 5 months to 15 years (2). Sanchis-Bielsa et al. (1) reported an average time to reaction of 7.1 years. In the series published by Lombardi et al. (8), the mean time to symptoms onset was 1.5 years, while Ficarra et al. (10) recorded a mean of 4.5 years. Other studies have described longer latency periods of 4 or 5 years.

Adverse reactions have been reported with practically all the products used. Silicone was the first product em-ployed on a large scale, and therefore accounts for most of the reported undesirable effects and the most virulent reactions. The term “siliconoma” has been used in reference to the orofacial granulomatous reactions produced by silicone infiltration.

Systemic toxicity has been suggested with some of these filler products, particularly silicone, since deposits of the material have been found in liver, spleen and kidneys after subcutaneous injection. However, no relation to connective tissue diseases or other disorders has been established.

Local toxicity includes pain, edema, ecchymosis, erythema, color changes, alterations in skin texture, overcorrection effects and local embolic phenomena. There have also been descriptions of extensive ulcerations that can affect bone, muscle and nerve structures (14).

The clinical manifestations include swelling or tumefaction of normally hard consistency. There have also been reports of reddening, burning sensation, tenderness in response to palpation and mild inflammation or even fibrous reactions. Sanchis-Bielsa et al. (1) found most patients to be asymptomatic – the reason for consultation being the presence of subcutaneous nodules, inflammation and deformation. They recorded no serious consequences in their patients, though there were cases of important facial deformity, epidermal necrosis or extensive deformation after subcutaneous silicone infiltration.

The histological findings are characteristic, with giant multinucleated cells reflecting foreign body reactions. Silicone infiltrations usually show abundant macrophages with intracytoplasmic vacuoles (15). Other products in turn induce classical foreign body granulomas in which fibrotic phenomena are also observed, along with violet-colored material deposits, asteroid bodies and histiocyte infiltrates (16).

The differential diagnosis should include erysipela, allergic contact dermatitis, facial edema with eosinophilia, apostematous glandularis cheilitis, Ascher syndrome, orofacial granulomatosis, Melkersson-Rosenthal syndro-me, sarcoidosis, cutaneous leishmaniasis, leprosy or tuberculosis. Cases manifesting as well defined lip nodules are suggestive of salivary gland tumors or cysts, such as mucoceles, as well as soft tissue tumors and cysts (2,10,12).

Etiological treatment (i.e., removal of the injected material) poses difficulties. Symptomatic treatment has inclu-ded local and systemic corticosteroids (prednisolone 1 mg/kg/day during 4-6 weeks, followed by gradual dose reduction). Corticosteroid injections inhibit fibroblast activity and the depositing of collagen, macrophage activity and the formation of giant cells – avoiding pain and swelling in most cases. However, corticosteroids can produce surrounding skin atrophy secondary to the inhibition of fibroblasts and keratinocyte activity. These side effects are dose-dependent, and skin atrophy in many cases is unavoidable (17).

Minocycline has yielded favorable results as a result of its antiinflammatory and immune modulating properties and anti-granulomatous effects, as demonstrated in vitro.

Allopurinol has also been used in the treatment of sarcoidosis and granulomas related to the use of cosmetic filler materials. In turn, while broad-spectrum antibiotic use has been reported, its efficacy has been questioned (12,18,19).

Other treatment proposals include 5% imiquimod (2,20) and hyaluronidase in the case of granulomas secondary to hyaluronic acid infiltrations (21,22).
